# Supplementary material for: Metabolic Profiles and Blood Biomarkers to Discriminate between Benign Thyroid Nodules and Papillary Carcinoma, Based on UHPLC-QTOF-ESI+-MS Analysis
Source: Int J Mol Sci. 2024 Mar 20;25(6):3495. doi: 10.3390/ijms25063495 (PMC10970441; doi:10.3390/ijms25063495)
Supplement: Supplementary file 1 [file ijms-25-03495-s001.zip › Suppl. Figure S1.pdf]

METABOLOMICS METHODOLOGY

Aim: metabolic profile of blood, urine by **untargeted** Metabolomics using succesive steps

Sample preparation

Extraction of metabolites with specific solvents

Data acquisition

High performance liquid chromatography coupled with Mass spectrometry (UHPLC-MS). Acquisition of chromatograms (RT-retention time, [M+1] *vs* PI-Peak Intensity)

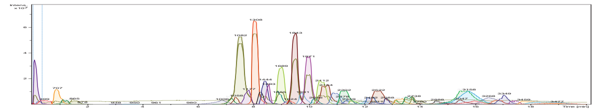

Data processing

Software Data Analysis 4.2-Find Molecular features (Bruker)
Filtration of data (RT alignment, S/N<5, PI<1000)

MATRIX: peak intensity = f(m/z)

Post-processing Statistics

Multivariate (T-test, PLSDA, Heatmaps, Random Forest, ROC curves) & Univariate ANOVA analysis using Metaboanalyst 5.0 (https://www.metaboanalyst.ca/MetaboAnalyst/)

Molecules Identification

Identification of putative biomarkers based on precursor [M+1] values
by Comparison with international databases
(Human Metabolomic Database, Lipidmaps, Pubchem)

Fig. S1. Metabolomics methodology. The steps from sample preparation to molecule identification.
