# Supplementary material for: Metabolic Profiles and Blood Biomarkers to Discriminate between Benign Thyroid Nodules and Papillary Carcinoma, Based on UHPLC-QTOF-ESI+-MS Analysis
Source: Int J Mol Sci. 2024 Mar 20;25(6):3495. doi: 10.3390/ijms25063495 (PMC10970441; doi:10.3390/ijms25063495)
Supplement: Supplementary file 1 [file ijms-25-03495-s001.zip › Suppl. Figure S2.pdf]

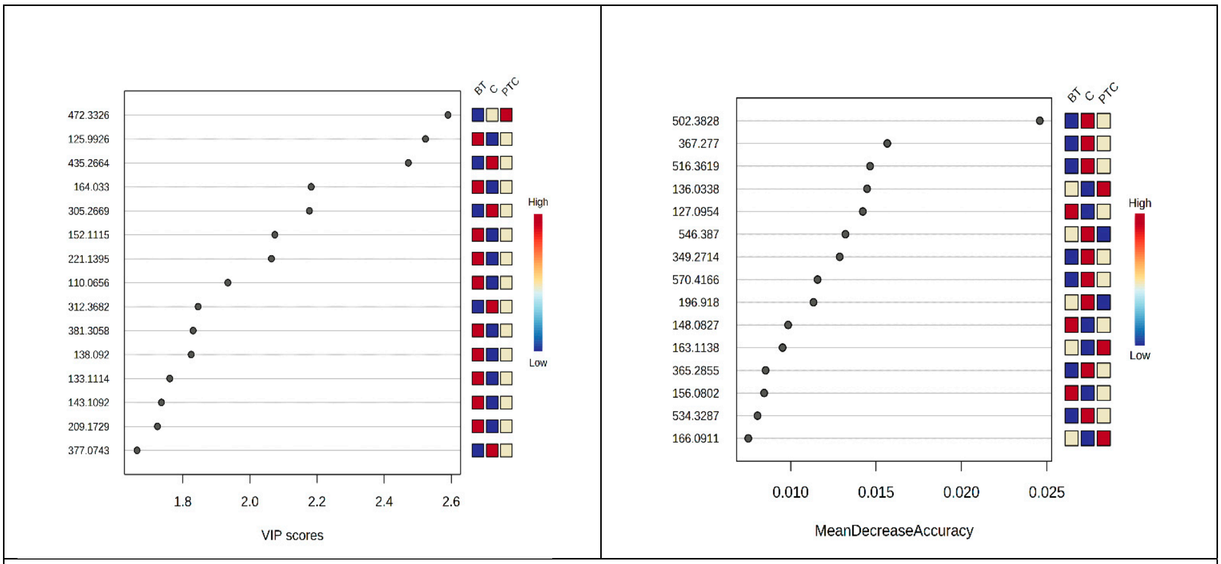

Fig. S2.A. VIP scores and Mean Decrease Accuracy according to Random Forest analysis for the 3 groups of patients: thyroid cancer (PTC), benign nodules (BT) comparative to healthy controls (C).

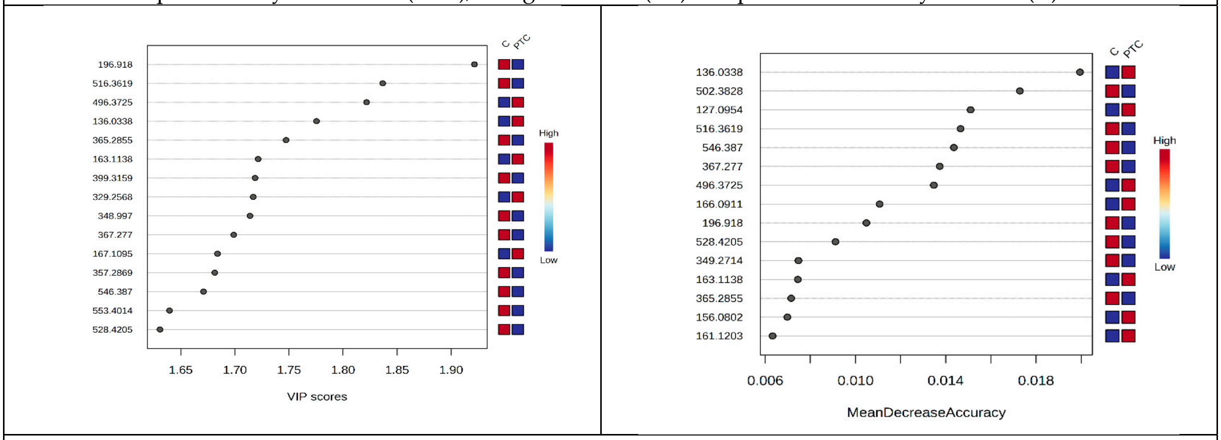

Fig. S2.B. VIP scores and Mean Decrease Accuracy according to Random Forest analysis to differentiate the metabolomic profile of thyroid cancer (PTC) patients comparative to healthy controls (C).

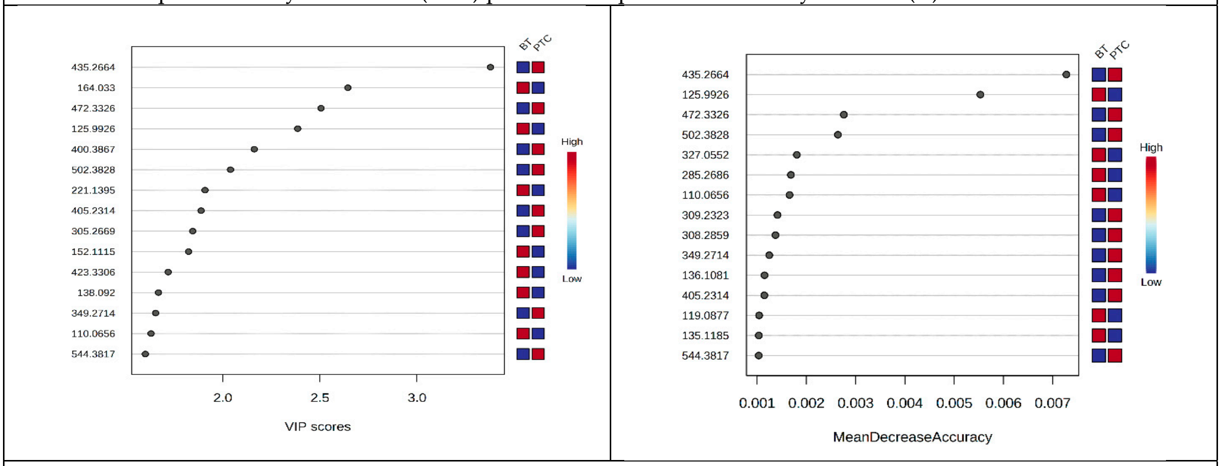

Fig. S2.C. VIP scores and Mean Decrease Accuracy according to Random Forest analysis) to differentiate the metabolomic profile of patients diagnosed with thyroid cancer (PTC) vs benign nodules (BT).
