# Supplementary material for: Metabolic Profiles and Blood Biomarkers to Discriminate between Benign Thyroid Nodules and Papillary Carcinoma, Based on UHPLC-QTOF-ESI+-MS Analysis
Source: Int J Mol Sci. 2024 Mar 20;25(6):3495. doi: 10.3390/ijms25063495 (PMC10970441; doi:10.3390/ijms25063495)
Supplement: Supplementary file 1 [file ijms-25-03495-s001.zip › Suppl. Table S1.pdf]

Table S1. Molecules (n=166) separated using HPLC-QTOF-ESI$^+$-MS and identified, according to their m/z values. The experimental m/z values were compared with the average of theoretical m/z values from the International database HMDB (Human Metabolomic DataBase). The accuracy of (theoretical – experimental) m/z values was below 20 ppm. The ID HMDB codes are mentioned in column 3.

m/z	Identification	ID (HMDB)
**TCA metabolites (n=7)**		
117.0930	Fumaric acid	HMDB0000134
119.0877	Succinic acid	HMDB0000254
135.1185	L-malic acid	HMDB0000156
161.1203	Tryptamine	HMDB0000303
177.0599	N-Hydroxyl tryptamine	HMDB0255154
177.1664	Serotonin	HMDB0000259
193.0031	Citric acid	HMDB0000094
**Amino acids and derivatives (n=32)**		
110.0656	Hypotaurine	HMDB0000965
116.1090	L-Proline	HMDB0000162
118.0886	Valine	HMDB0000883
120.0826	Threonine	HMDB0000167
125.9926	Taurine	HMDB0000251
130.0882	Oxoproline	HMDB0304793
131.1090	Ketoleucine	HMDB0000695
132.1046	Isoleucine	HMDB0000172
133.1114	Asparagine	HMDB0000168
138.0920	Tyramine	HMDB0000306
144.0896	Proline betaine	HMDB0004827
147.0221	Lysine	HMDB0000182
147.1168	L-Glutamine	HMDB0000641
148.0827	Glutamic acid	HMDB0000148
148.9852	D-2-Hydroxyglutaric acid	HMDB0000606
156.0802	Histidine	HMDB0000177
158.9969	N-Acetylproline	HMDB0094701
163.6470	5-hydroxylysine	HMDB0000450
166.0911	Phenylalanine	HMDB0000159
170.0280	1-Methylhistidine	HMDB0000001
173.1574	Glycylproline	HMDB0000721
174.0451	Arginine	HMDB0000517
175.1360	Argininic acid	HMDB0003148
176.0426	Tyrosine	HMDB0000158
182.1604	L-Tryptophan	HMDB0000929
205.0995	Kynurenine	HMDB0000684
209.1729	Glycyl-Histidine	HMDB0028843
213.1822	Acetyl hydroxytryptamine	HMDB0001238
219.1772	5-Hydroxy-L-tryptophan	HMDB0000472
221.1395	N-Acetyl-L-tyrosine	HMDB0000866

224.1901	L-Cystine	HMDB0000192
241.1794	Glutathione	HMDB0062697
**Butyric derivatives, Methionine& Cysteine Selenocomplexes (n=10)**		
104.1090	GABA	HMDB0000112
105.0350	3-Hydroxybutyric acid	HMDB0000357
121.1030	(S)-3,4-Dihydroxybutyric acid	HMDB0000337
136.0338	Homocysteine	HMDB0000742
150.1149	Methionine	HMDB0000696
164.0330	Acetylcysteine	HMDB0001890
172.1007	L-Homocysteine sulfate	HMDB0002238
202.2204	Cysteine-S-sulfate	HMDB0000731
183.1683	Methylselenocysteine	HMDB0004113
196.9180	Selenomethionine	HMDB0003966
**Purines and pirimidines (n=27)**		
113.0656	Uracil	HMDB0000300
115.1136	Dihydrouracil	HMDB0000076
127.0954	Thymine	HMDB0000262
128.9876	2-Thiouracil	HMDB0245323
129.1295	Dihydrothymine	HMDB0000079
136.1081	Adenine	HMDB0000034
137.1347	Hypoxanthine	HMDB0000157
143.1092	5-Hydroxymethyluracil	HMDB0000469
152.1115	Hydroxy adenine	HMDB0000403
167.1095	3-Methylxanthine	HMDB0001886
171.1521	Propylthiouracil	HMDB0014690
243.1637	Thymidine	HMDB0000273
253.0018	Deoxyinosine	HMDB0000071
253.1846	Deoxyadenosine	HMDB0000101
259.2055	3-Methyluridine	HMDB0004813
268.2171	Adenosine	HMDB0000050
269.2104	Inosine	HMDB0000195
284.3360	Guanosine	HMDB0000133
285.1756	Xanthosine	HMDB0000299
298.2136	1-Methylguanosine	HMDB0001563
312.3682	N2,N2-Dimethylguanosine	HMDB0004824
325.2580	Uridine 5'-monophosphate	HMDB0000288
348.9970	Adenosine monophosphate	HMDB0000045
349.2714	Inosinic acid	HMDB0000175
364.9703	Guanosine monophosphate	HMDB0001397
399.3159	S-Adenosylmethionine	HMDB0001185
405.2314	Uridine 5'-diphosphate	HMDB0000295
**Free Fatty Acids (n=14)**		
229.1460	Myristic acid C14:0	HMDB0000806
255.0099	Palmitoleic acid C16:1	HMDB0003229

257.2524	Palmitic acid C16:0	HMDB0000220
279.2371	Linolenic acid C18:3	HMDB0003073
281.2529	Linoleic acid C18:2	HMDB0000673
283.2651	Oleic acid C18:1	HMDB0000207
285.2686	Stearic acid C18:0	HMDB0000827
305.2669	Arahidonic acid C20:4	HMDB0001043
307.2610	Eicosatrienoic acid C20:3	HMDB0002925
309.2323	Eicosadienoic acid C20:2	HMDB0005060
311.2621	Eicosenoic acid C20:1	HMDB0002231
313.2395	Arachidic acid C20:0	HMDB0002212
327.0552	Methyl arachidic acid C21:0	LMFA01020018
329.2568	Docosahexenoic acid C22:6	HMDB0002183
**AcylCarnitines (n=24)**		
162.1152	L-carnitine	HMDB0000062
204.1263	Propenoylcarnitine	HMDB0013124
216.2365	L-Acetylcarnitine	HMDB0000201
218.1722	Propionylcarnitine	HMDB0000824
230.2524	Butenylcarnitine	HMDB0013126
244.2682	Tiglylcarnitine	HMDB0002366
246.1526	Valerylcarnitine	HMDB0013128
274.2799	Glutaconylcarnitine	HMDB0013129
276.7343	Glutarylcarnitine	HMDB0013130
286.3161	Octenoylcarnitine	HMDB0013324
288.2957	9-Decenoylcarnitine	HMDB0013205
304.3065	3-hydroxydecanoyl carnitine	HMDB0061636
314.255	trans-2-Dodecenoylcarnitine	HMDB0013326
332.3381	Tetradecanoylcarnitine	HMDB0005066
342.3798	9,12-Hexadecadienoylcarnitine	HMDB0013334
372.3551	trans-Hexadec-2-enoyl carnitine	HMDB0006317
396.3551	9-Hexadecenoylcarnitine	HMDB0013207
398.3699	L-Palmitoylcarnitine	HMDB0000222
398.7684	Stearoylcarnitine	HMDB0000848
400.3867	Methyldocosanoylcarnitine (adduct)	HMDB0240952
428.3246	Arachidyl carnitine	HMDB0006460
439.3864	L-Octanoylcarnitine	HMDB0000791
456.3621	Pimelylcarnitine	HMDB0013328
472.3326	Cervonyl carnitine	HMDB0006510
**Bile acids (n=14)**		
**377.2260**	Lithocholic acid	HMDB0000717
**391.2990**	12-Ketodeoxycholic acid	HMDB0000328
**393.2220**	7-Ketodeoxycholic acid	HMDB0000391
**393.3200**	3-Oxocholic acid	HMDB0000502
**395.2470**	Deoxycholic acid glycine conjugate	HMDB0000631
**407.1900**	Lithocholyltaurine	HMDB0000722

**407.3180**	Taurodeoxycholic acid	HMDB0000896
**409.2400**	7-Methyl-cholic acid	HMDB0244296
**423.3310**	Ursocholic acid	HMDB0000917
**450.3120**	Norcholic acid	HMDB0255727
**484.3930**	Chenodeoxycholic acid	HMDB0000518
**500.3890**	Deoxycholic acid	HMDB0000626
**516.3620**	Taurocholic acid	HMDB0000036
**553.4010**	Lithocholic acid glucuronide	HMDB0002513
**Corticosteroids (n=8)**		
347.1934	Deoxycortisol	HMDB0004030
361.2327	Cortisone	HMDB0002802
363.2255	Cortisol	HMDB0000063
365.1439	Tetrahydrocortisone	HMDB0000903
365.2855	Dihydrocortisol	HMDB0003259
367.2770	Tetrahydrocortisol	HMDB0000949
377.0743	Oxocortisol	HMDB0000332
379.0897	Hydroxycortisol	HMDB0000418
**Prostaglandins (n=8)**		
335.1374	PGA2/B2	HMDB0002752
337.2411	PGA1	HMDB0002656
343.3139	Dinor-6-keto-PGF1a	HMDB0062618
351.2565	PGE3/D3	HMDB0002664
353.2739	PGE2 /D2	HMDB0060041
355.2895	PGF2a	HMDB0001139
357.2869	PGF1a	HMDB0002685
381.3058	Dimethyl-PGE2	HMDB0244730
**Phospholipids (n=22)**		
258.2841	Glycerophosphocholine	**HMDB0000086**
435.2664	LysoPA(18:2)	HMDB0007856
439.2300	LysoPA(18:0)	HMDB0007854
493.3107	LysoPA(22:1)	HMDB0114750
494.3330	LysoPC(16:1)	HMDB0010383
496.3725	LysoPC(16:0)	HMDB0010382
502.3828	LysoPE(20:4)	HMDB0011487
518.3312	LysoPC(18:3)	HMDB0010387
520.3492	LysoPC(18:2)	HMDB0010386
522.3648	LysoPC(18:1)	HMDB0002815
523.3199	LysoPA(24:0)	HMDB0114756
524.3817	LysoPC(18:0)	HMDB0010384
526.3022	LysoPE(22:6)	HMDB0011496
528.4205	LysoPE(22:5)	HMDB0011494
534.3287	LysoPE(22:2)	HMDB0011492
542.3328	LysoPC(20:5)	HMDB0010397
544.3817	LysoPC(20:4)	HMDB0010395

546.3870	LysoPC(20:3)	HMDB0010393
568.3494	LysoPC(22:6)	HMDB0010404
570.4166	LysoPC(22:5)	HMDB0010402
572.4492	LysoPC(22:4)	HMDB0010401
732.5668	PC(16:0/16:1)	HMDB0007969
