# Supplementary figures and images for: Metabolic Profiles and Blood Biomarkers to Discriminate between Benign Thyroid Nodules and Papillary Carcinoma, Based on UHPLC-QTOF-ESI+-MS Analysis
Source: Int J Mol Sci. 2024 Mar 20;25(6):3495. doi: 10.3390/ijms25063495 (PMC10970441; doi:10.3390/ijms25063495)

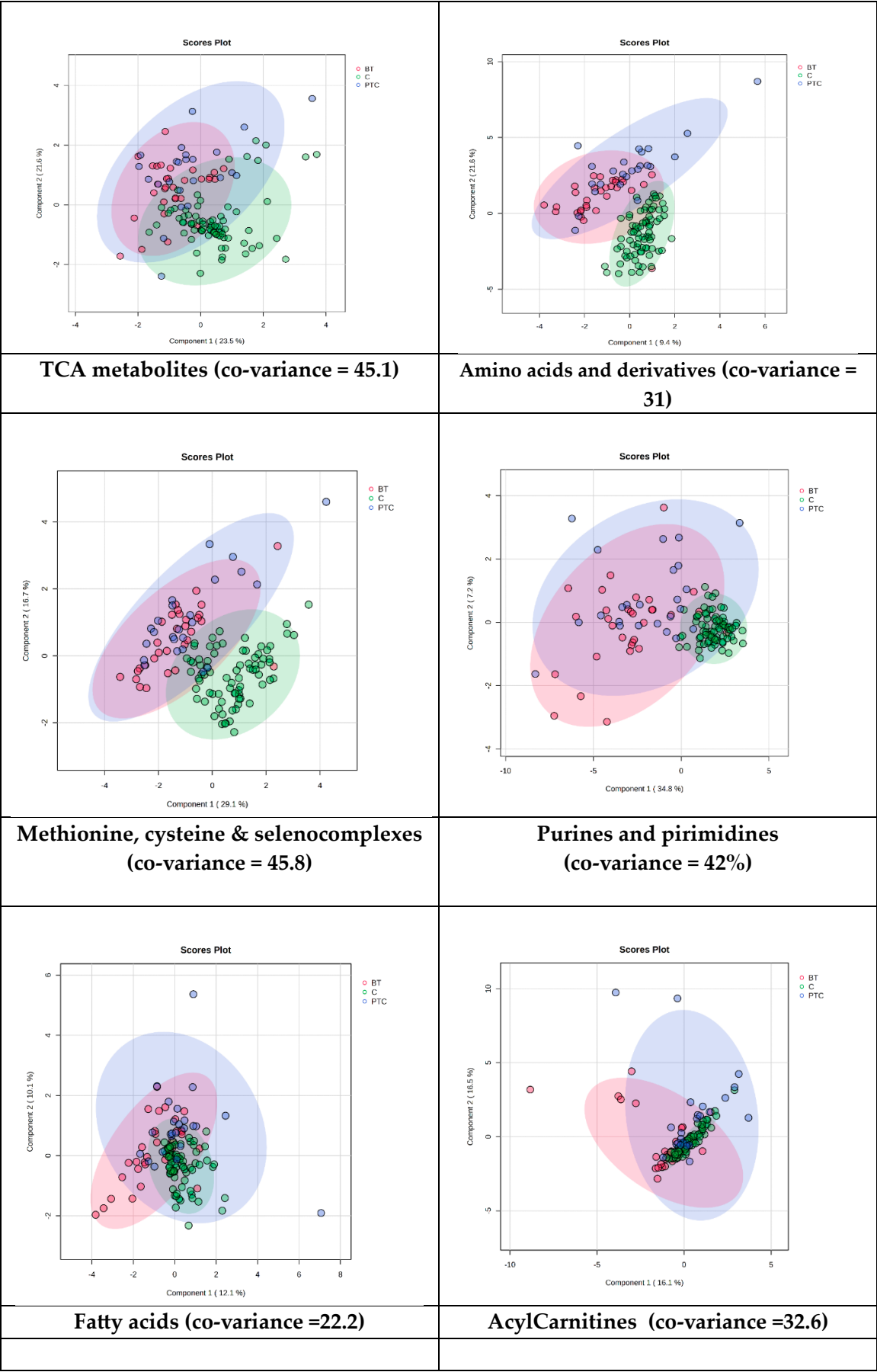

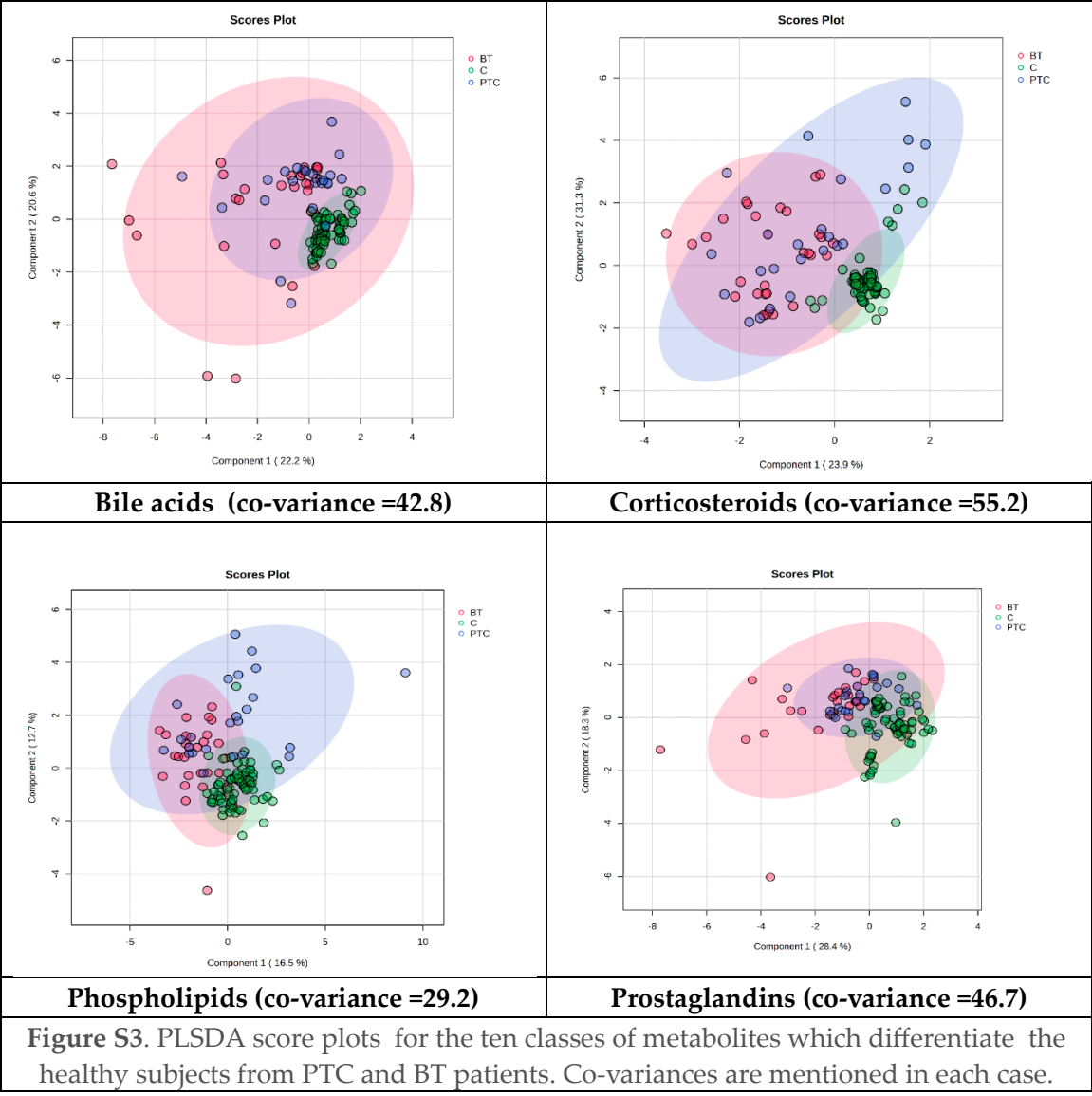

Supplement: Supplementary file 1 [file ijms-25-03495-s001.zip › Suppl. Figure S3.pdf]

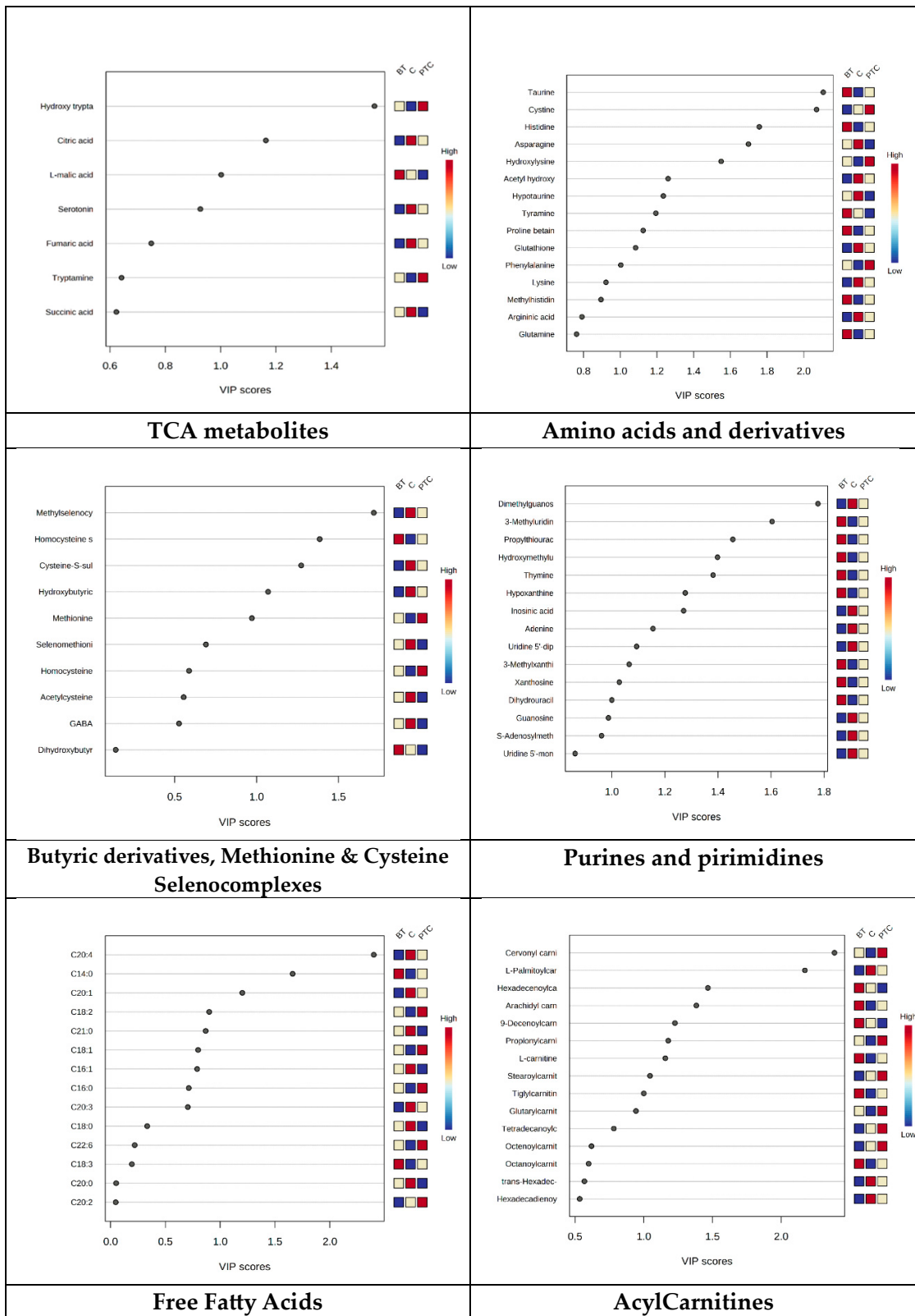

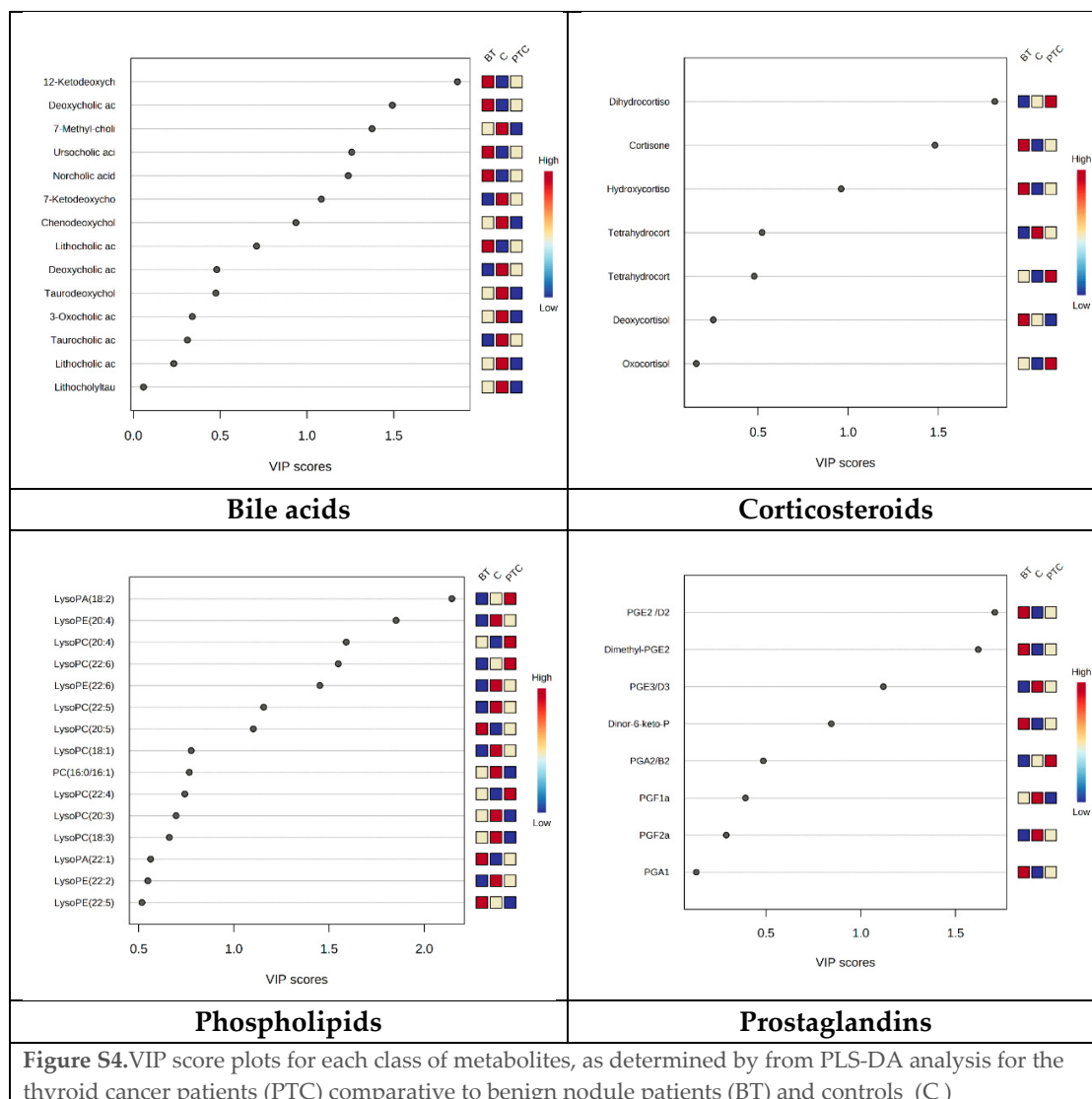

Supplement: Supplementary file 1 [file ijms-25-03495-s001.zip › Suppl. Figure S4.pdf]
